# Physicochemical Investigations of Chitosan-Based Hydrogels Containing *Aloe Vera* Designed for Biomedical Use

**DOI:** 10.3390/ma13143073

**Published:** 2020-07-09

**Authors:** Anna Drabczyk, Sonia Kudłacik-Kramarczyk, Magdalena Głąb, Magdalena Kędzierska, Anna Jaromin, Dariusz Mierzwiński, Bożena Tyliszczak

**Affiliations:** 1 Faculty of Materials Engineering and Physics, Institute of Materials Science, Cracow University of Technology, 37 Jana Pawła II Av., 31-864 Krakow, Poland; magdalenaglab@op.pl (M.G.); dariusz.mierzwinski@pk.edu.pl (D.M.); 2 Department of Chemotherapy, Medical University of Lodz, WWCOiT Copernicus Hospital, 90-001 Lodz, Poland; kameleonmagda@gmail.com; 3 Department of Lipids & Liposomes, Faculty of Biotechnology, University of Wroclaw, 14a Joliot-Curie, 50-383 Wroclaw, Poland; anna.jaromin@uwr.edu.pl

**Keywords:** hydrogels, chitosan, *Aloe vera*, tensile strength, MTT reduction assay, cytotoxicity, surface morphology, composite polymers

## Abstract

In this work, synthesis and investigations on chitosan-based hydrogels modified with *Aloe vera* juice are presented. These materials were synthesized by UV radiation. Investigations involved analysis of chemical structure by FTIR spectroscopy, sorption properties in physiological liquids, strength properties by texture analyzer, surface topography by Atomic Force Microscopy (AFM technique), and in vitro cytotoxicity by MTT test using L929 murine fibroblasts. Particular attention was focused both on determining the impact of the amount and the molecular weight of the crosslinker used for the synthesis as well as on the introduced additive on the properties of hydrogels. It was proven that modified hydrogels exhibited higher swelling ability. Introduced additive affected the tensile strength of hydrogels—modified materials showed 23% higher elongation. The greater amount of the crosslinker used in the synthesis, the more compact the structure, leading to the lower elasticity and lower sorption of hydrogels was reported. Above 95%, murine fibroblasts remained viable after 24 h incubation with hydrogels. It indicates that tested materials did not exhibit cytotoxicity toward these lines. Additionally, materials with *Aloe vera* juice were characterized by lower surface roughness. Conducted investigations allowed us to state that such modified hydrogels may be considered as useful for biomedical purposes.

## 1. Introduction

Over the last few decades, the interest in hydrogels has increased due to their unique properties. One of the most important ones is biocompatibility, which means that in contact with blood, body fluids, or living tissues, these materials do not show negative effects on the immune system and are characterized by the lack of toxicity [1]. Among many areas, particular interest in hydrogels is observable in tissue engineering. Studies on the hydrogels with potential application in this area of medicine were performed by Boso et al. [2], Mantha et al. [3], and Chung et al. [4]. Interesting investigations were performed by Ilgin et al., who described the synthesis of hydrogels based on *N*-tert-butylmaleimic acid (TBMAC) and poly(*N*-isopropylcrylamide) (p(NIPAM)). It was proven that these hydrogels changed their properties under the influence of pH and temperature. This dual stimuli responsive material was obtained by free radical copolymerization in aqueous solution and considered for use in controlled drug delivery systems [5]. Timaeva et al. proposed the development of hydrogels based on a nontoxic biocompatible polymer, i.e., poly-*N*-vinylpyrrolidone (PVP), with rare earth elements (RE), which, according to the recent reports, show antibacterial properties. The obtained PVP/RE composite may be used in antibacterial therapy [6]. Semi-interpenetrating networks (semi-IPN) hydrogels were developed by García-Fernández et al. The materials were based on gelatin and hyaluronic acid with the addition of anti-inflammatory drugs. Such hydrogels can be used to treat arthritis [7]. A very interesting solution is the idea of Chouhan et al. concerning the hydrogel eye drop as a carrier providing and maintaining medicines on the eye surface. In these studies, the use of gellan fluid gel to ensure a continuous supply of drugs was presented [8]. Another important solution was reported by Tamahkar et al., who presented multilayer hydrogel dressings. The materials consisted of carboxylated poly(vinyl alcohol), gelatin, and hyaluronic acid. The upper layer constituted a physical barrier for microorganisms and was responsible for humidity control. The task of the lower layer was to remove exudate from the wound and to control the release of antibiotics placed in the middle layer. The multilayer hydrogels obtained showed a high application potential [9].

Hydrogel materials, which were initially used mainly as contact lenses [10], are now used in a very wide range of fields [11], i.e., in medicine, e.g., for delivering biotherapeutic molecules [12], tissue engineering [13], or in preparation of wound dressings [14]. Moreover, hydrogels based on natural resources are very often used in cosmetology [15]. These polymers find also the application in agriculture, i.e., in irrigation systems [16] or as absorbers of pollutants [17].

In recent years, special attention has been paid to the materials of natural origin and such an upward trend was also observed in the synthesis of hydrogels [18]. One of the frequently used substrates in the synthesis of hydrogels is chitosan [19]. Chitosan is an organic compound belonging to the group of polysaccharides. It is mainly obtained by a deacetylation of chitin [20]. It is nontoxic and biocompatible [21]. This polysaccharide has been used by Venkatesan et al. for the synthesis of hydrogels containing, additionally, carbon nanotubes and such materials have been analyzed in view of their antimicrobial activity [22]. Another solution was presented by Ziminska et al. They prepared hydrogel polymers based on chitosan-grafted poly(*N*-isopropylacrylamide) and proved that such materials may be used for sustained delivery of genes [23]. Other frequently used natural polymers in the preparation of hydrogel materials are starch [24], gelatin [25], hyaluronic acid [26], and alginates [27,28]. For example, Meng et al. described lignin-based hydrogels, which were by-products of the pulp and paper industry and were selected for their biocompatibility, biodegradability, and nontoxicity characteristics. Lignin-based hydrogels can serve as absorbers for heavy metal ions as well as biosensors and intelligent materials [29]. Moreover, hydrogels based on chitosan are widely used as dressing materials. They are mainly intended for difficult-to-heal wounds, such as those after surgery, burns, or ulcers [30]. In order to improve selected properties of hydrogels, a common solution is to combine different types of natural polymers. Taira et al. developed the method of electrochemical printing of algine-gel hydrogel with potential application in the creation of three-dimensional tissue organs [31]. Gilarska et al. focused on the obtaining ternary systems containing collagen/chitosan/hyaluronic acid. The obtained hydrogels can be widely used in bone regeneration procedures [32].

Various types of substances are often added to the hydrogel materials in order to give new properties or improve the existing ones [33]. Among such modifiers, *Aloe vera* extract can be mentioned. *Aloe vera* is recognized as a medicinal plant known and used worldwide [34]. Due to its chemical composition, i.e., the presence of many active compounds, this substance is used in the treatment of various ailments, e.g., inflammations, difficult-healing wounds, ulcers, or various skin diseases [35]. Anthraquinones and glycosides present in *Aloe vera* juice are responsible for its antioxidant and anti-inflammatory properties [36]. In recent years, the number of applications of *Aloe vera* extract has been increased both in medicine and cosmetology [37]. Silva et al. received sponges based on *Aloe vera* gel with a thin layer of gellan gum. Synthesized sponges were characterized by good mechanical properties and lack of toxicity. The results of their investigations indicated that these materials can be used in regenerative medicine [38]. In tissue engineering, materials developed by Bhaarathy et al. maybe also mentioned. They proposed composites consisting of poly(l-lactic acid)-*co*-poly (ε-caprolactone) (PLACL), silk fibroin (SF), and *Aloe vera* (AV) that are obtained by electrospinning. *Aloe vera* was used due to its anti-allergic and anti-inflammatory properties [39].

Difficult-healing wounds are still problematic and require the development of specific dressings that provide an adequate environment for the healing process. Hydrogels, due to their properties, may be applied as such dressings. The novelty of the research is the development of adequate dressing materials with desirable properties including suitable sorption properties and the tensile strength. Such a material should provide the environment that promotes the wound-healing process, i.e., it should absorb the wound exudate, adhere well to the wound, and—depending on the location of the wound—exhibit adequate flexibility. These properties may be achieved by chitosan-based hydrogels containing *Aloe vera* juice. Due to the presence of hydrophilic functional groups in the polymer network of hydrogels, these polymers exhibit sorption ability. Moreover, *Aloe vera* juice shows anti-inflammatory activity and such an additive may contribute to the soothing effect of hydrogels containing this modifying agent that may also affect positively on the wound healing.

In the presented studies, chitosan-based hydrogels were prepared via UV radiation. Next, physicochemical properties of the materials were characterized including swelling ability, tensile strength under the applied tension, or cytotoxicity toward selected cell lines. Swelling ability is important due to the previously mentioned absorption of the wound exudate by such a material. Otherwise, such an exudate might accumulate near the wound and impede the healing process. A significant aspect of the investigations was to determine the impact of the amount and the molecular weight of the crosslinking agent used for the synthesis of hydrogels on their physicochemical properties. The amount of the crosslinker used influences the crosslinking density of the formed hydrogel. This, in turn, may affect their swelling properties and tensile strength. Tensile strength of the dressing material is important for application reasons. When such a dressing is applied on the wound in a place of high mobility (e.g., elbow, knee, etc.) then the elasticity of the dressing is preferable. Otherwise, it would be difficult to maintain such a dressing. In other cases, the stiffer dressing may be more preferable. Therefore, it is essential to determine the mentioned properties.

## 2. Materials and Methods

### 2.1. Materials

Crosslinking agent, i.e., diacrylate poly(ethylene glycol) (PEGDA; average molecular weight Mn = 575 g/mol—PEGDA 575 and Mn = 700 g/mol—PEGDA 700) and photoinitiator, i.e., 2-hydroxy-2-methylpropiophenone (97%, *d* = 1.077 g/mL) were received from Merck (Darmstadt, Germany). Chitosan (low molecular weight, deacetylation degree 75–85%) was received from Sigma Aldrich (Saint Louis, MO, USA). *Aloe vera* juice (99.5%) was bought in Herbal Pharmaceuticals (Kraków, Poland).

### 2.2. Synthesis of Hydrogels

Hydrogels were obtained by photopolymerization using EMITA VP-60 lamp (power: 180 W, λ = 320 nm, Famed, Lodz, Poland) as a radiation source. For this purpose, the initial solution, i.e., 1% solution of chitosan in 0.05% acetic acid, was prepared. This solution (50 mL) was mixed with an appropriate amount (2, 4, 8, 10, 12 mL) of crosslinking agent (PEGDA 575) and 0.5 mL of photoinitiator (2-hydroxy-2-methylpropiofenone). The prepared solutions were stirred intensively to obtain homogeneous mixtures, then poured into the Petri dishes, and treated with UV radiation for 120 s.

Next, the second series of syntheses, i.e., with PEGDA 700, was conducted in a similar way. Adequate amounts of the chitosan solution, crosslinker, and the photoinitiator were mixed and treated with UV radiation for 120 s.

The compositions of obtained samples are presented in Table 1 and Table 2.

For the materials obtained, the tests to determine the physicochemical properties, such as, e.g., swelling ability or strength properties, were performed. The favorable parameters of synthesis were selected and hydrogel materials modified with *Aloe vera* were obtained. Procedure of preparation of hydrogels with *Aloe vera* juice was analogous. The only difference was that the reaction mixture subjected to the UV radiation, apart from the chitosan solution, crosslinking agent, and the photoinitiator, consisted also of the adequate amount of *Aloe vera* juice. The whole mixtures were also subjected to UV radiation for 120 s.

The composition of modified hydrogels is presented in Table 3.

**Figure 1:**
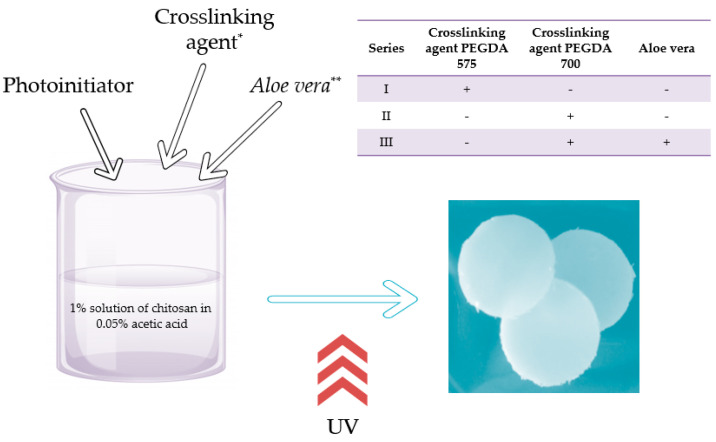
The scheme of the hydrogels’ preparation (*: PEGDA 575 or PEGDA 700 crosslinker; **: *Aloe vera* addition in the case of series III).

After the synthesis, prepared hydrogels were immersed in PBS (phosphate buffered saline) for 15 min to elute all unreacted reagents from the polymer networks. Such an operation was repeated three times. The procedure of preparation of hydrogels scheme is schematically presented in Figure 1.

Next, hydrogels were dried in the air at room temperature for 48 h and subjected to the investigations.

Chemical structure, sorption, strength, and roughness properties were characterized. Additionally, the cytotoxic properties of the obtained materials were determined.

### 2.3. Methodology of Measurements

#### 2.3.1. Analysis of the Chemical Structure of Hydrogels

Fourier transform infrared spectroscopy (FTIR) analysis was performed to determine functional groups present in the analyzed hydrogel materials. Spectrum 65 (Perkin Elmer, Waltham, MA, USA) spectrometer equipped with attenuated total reflection (ATR) attachment with a diamond/ZnSe crystal was used for this purpose. FTIR spectra were obtained within the range of 4000–600 cm^−1^ (32 scans at 4.0 cm^−1^ resolution). The study was performed in room temperature.

#### 2.3.2. Sorption Properties of Hydrogels

The absorbency of fluids by hydrogels is expressed by the ratio of the weight of fluid absorbed by the sample to the weight of the sample in the dry state. The analysis consisted of the immersing of 1 g of dry hydrogel in a sterile vessel containing 50 mL of a suitable liquid for a certain period of time. Next, the sample was separated from the liquid and weighed. The analysis of sorption properties of hydrogels in distilled water and hemoglobin (2% aqueous solution of pork hemoglobin) was performed. The samples were weighed after 1 h, 24 h, and 72 h. The swelling ratio was determined for the materials tested according to the formula below (1):

(1) $$\alpha =\frac{m-m_{0}}{m_{0}}$$

where α is the swelling ratio, g/g; m is the weight of swollen hydrogel, g; and m_0_ is the weight of dry hydrogel, g.

#### 2.3.3. Mechanical Properties of Hydrogels

In order to determine the mechanical properties of hydrogels, a strength analysis was conducted. Mechanical studies were performed in accordance with ISO 527-2 type 5A and ISO 37 type 2. For this purpose, the paddle-shaped samples using ZCP020 blanking were cut and placed in the jaws of the Brookfield CT3 texture analyzer. As a result of the analysis, the dependence of stress on strain was determined. The study allowed us to characterize the tensile strength and the percentage elongation of the materials tested. The tensile strength (Rm) was determined using formula (2) and the percentage elongation (A) using formula (3) below.

(2) $$\mathrm{Rm}=\frac{\mathrm{Fm}}{S_{0}}$$

(3) $$A=\frac{100\times \left(l_{u}-l_{0}\right)}{l_{0}}$$

where Fm is the maximum strength, S_0_ is the cross-sectional area of the sample in its initial state, l_u_ is the measuring length after sample rupture, and l_0_ is the measuring length of the sample in its initial state.

Analysis of the mechanical properties allowed selecting hydrogels for further research. Studies on the materials that were characterized by too high brittleness were not possible. Therefore, such materials were not subjected to further analyses.

#### 2.3.4. Morphological Properties of Hydrogels

The surface topography of samples was characterized using atomic force microscopy (AFM). Analysis was performed using Bruker Atomic Force Microscope (Billerica, MA, USA), FastScan head type. Measurement range: XY: 30 μm, Z: 3 μm. Measurement mode: PEAKFORCE QNM SCM using tube T (f: 75 kHz, k: 2.8 N/m, length: 225 μm). A study was also conducted to determine the impact of *Aloe vera* addition on the topography of the hydrogel materials. Images presented in the article are representative of the whole surface of the hydrogels.

#### 2.3.5. Cytotoxicity of Hydrogels

Materials with potential medical use are subjected to in vitro biological tests. Therefore, the investigations on the cytotoxicity of materials to selected cells was also performed. The study is of the key importance in assessing the safety of materials and the possibility of investigating them using more advanced biological tests, including in vivo ones. One of the commonly used tests to determine cytotoxic properties is the MTT reduction assay. This test is based on the monitoring of the reactions of the culture cells after exposure to the tested substances. In the MTT test, the cell viability is determined by characterizing the metabolic activity of the cells. It is possible, by determining the activity of mitochondrial dehydrogenase, to convert the soluble salt of tetrazol (3-(4,5-dimethyl-thiazol-2-yl)-2,5-diphenyletrazol bromide) (MTT reagent) to insoluble formazan, which is a dark blue product of the above reaction. The resulting formazan crystals are then dissolved in DMSO or isopropanol and the color intensity of the resulting solution is determined spectrophotometrically in the wavelength range of 492–570 nm. Cell viability is determined by the proportionality of the amount of reduced MTT to the oxidative activity of cellular mitochondria. In the study, L929 mouse fibroblasts obtained from American Type Cell Culture Collection (Rockville, MD, USA) were used. The cells were incubated in the tissue culture flasks on RPMI-1640 medium supplemented with antibiotics, i.e., penicillin (100 U/mL) and streptomycin (100 µg/mL) as well as with inactivated bovine serum (10 wt%; Cytogen, Zgierz, Poland) under standard conditions (37 °C, 5% CO_2_, > 90% humidity). For cytotoxicity tests, a suspension of fibroblasts of L-929 line with concentration of 2 × 10^5^ cells/mL was prepared. Then, 100 µL of the cell suspension were placed in each well of 96-well platelet and incubated for 24 h (under standard conditions). Hydrogel samples of 1/10 of the well area were prepared and placed in the medium (5 mL). Next, they were placed in the appropriate wells of the plate. Eight samples of each hydrogel were tested. The following control samples were also prepared: K (+) viability control (cell culture without hydrogel) and K (−) cytotoxicity control (cells incubated with 1% phenol solution, a compound characterized by the strong cytotoxicity to cells). The plates were incubated for 24 h under standard conditions. After incubation, the substrate was removed from the plates and then replaced with 100 μL of fresh one. Then, 20 μL of MTT reagent (Merck; concentration: 5 mg/mL) was introduced into each well and the plates were incubated for 4 h (under standard conditions). After incubation, the plate was centrifuged (1200 rpm, 10 min) and the supernatant was removed from the cells. The formazan crystals were dissolved in 150 µL DMSO and 25 µL glycine buffer and incubated at room temperature for 15 min. Then, 160 µL of liquid were taken from each well and transferred to a new 96-well plate. Absorbance was measured using Spectramax multi-detection reader (Thermo Fisher Scientific, Waltham, MA, USA), applied wavelength: 570 nm.

## 3. Results and Discussion

### 3.1. Results of Syntheses Performed with Crosslinking Agent with Different Amounts and Molecular Weights

In Table 4 and Table 5 observations concerning the course of the crosslinking process are presented.

As it can be seen in Table 5, introduction of *Aloe vera* juice into the reaction mixture resulted in such dilution of this mixture that 8 mL of crosslinker was not enough to receive properly crosslinked hydrogel material. Therefore, only two types of modified hydrogels were obtained, i.e., those with 10 and 12 mL of crosslinking agent (PEGDA 700).

The obtained materials were subsequently subjected to the analyses aimed at determining their physicochemical and biological properties.

### 3.2. Chemical Analysis of Hydrogels

**Figure 2:**
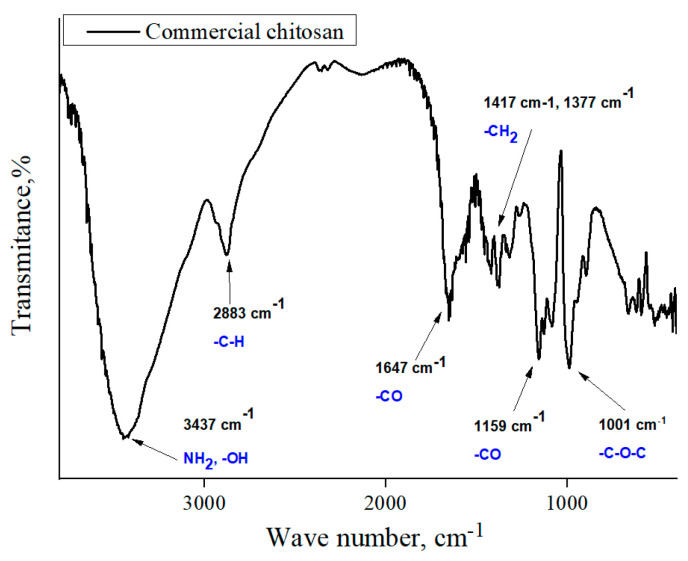
FTIR spectrum of commercial chitosan.

**Figure 3:**
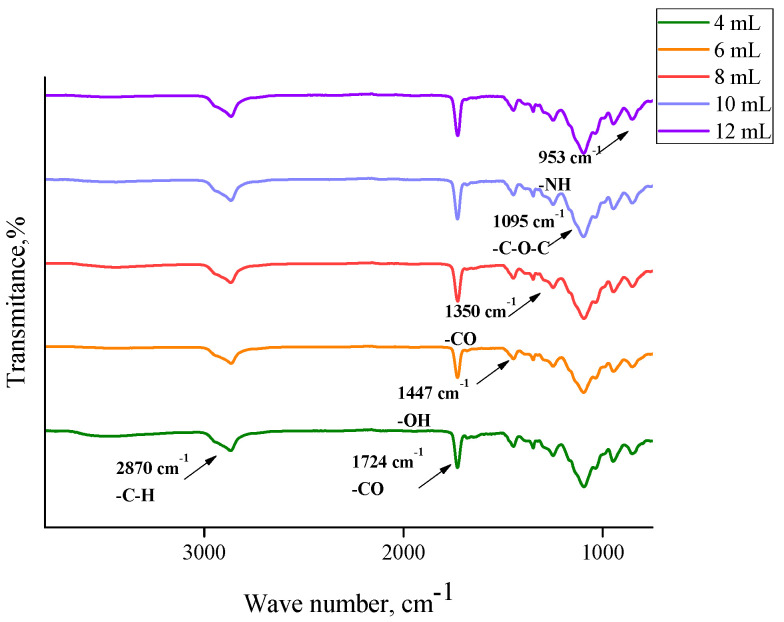
FTIR spectra of materials with different contents of crosslinking agent PEGDA 575.

**Figure 4:**
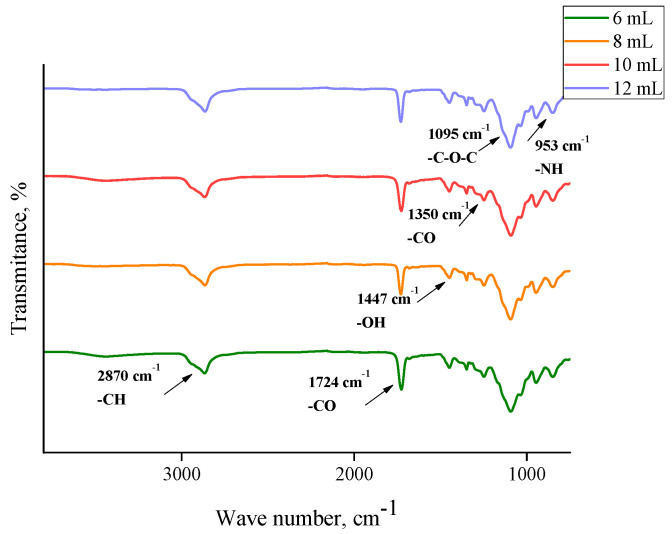
FTIR spectra of materials with different contents of crosslinking agent PEGDA 700.

**Figure 5:**
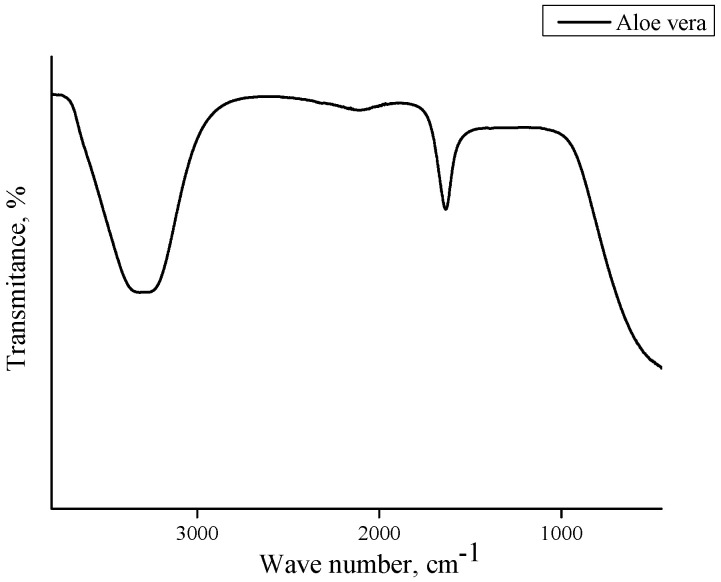
FTIR spectrum of *Aloe vera* juice.

**Figure 6:**
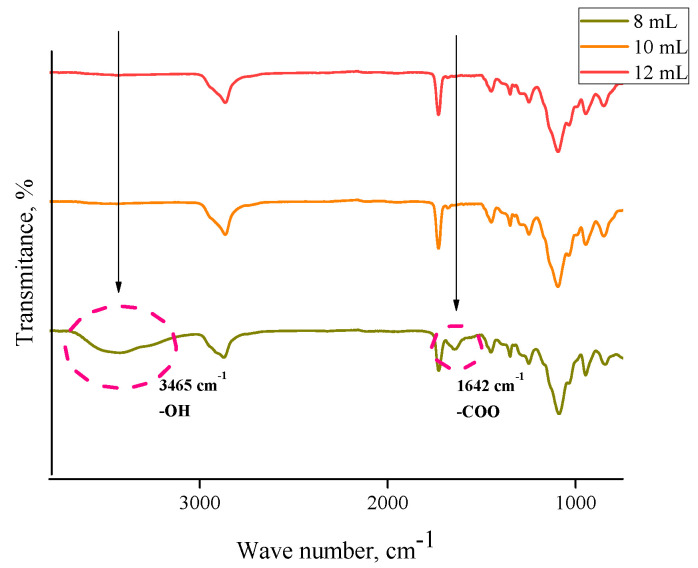
FTIR spectra of materials with different contents of crosslinking agent PEGDA 700 and 10 mL of *Aloe vera* juice.

In order to determine the functional groups present in the hydrogels, FTIR analysis was performed. This study was also performed for commercial chitosan (that was used for the preparation of hydrogels) and the *Aloe vera* juice to compare the spectra of these reagents with the spectra of hydrogels based on them. Obtained FTIR spectra are shown in Figure 2, Figure 3, Figure 4, Figure 5 and Figure 6.

In the above FTIR spectra, characteristic absorption bands from functional groups of chitosan can be observed, e.g., the absorption band observed at 2870 cm^−1^ derived from the stretching vibrations of the C–H bond occurring in this polysaccharide. Next, stretching vibrations of C=O group at wavelength number 1724 cm^−1^ may have originated from PEGDA present in crosslinked hydrogel matrix. The low intensity absorption band at 1447 cm^−1^ came from the hydroxyl group of chitosan. The band at 1350 cm^−1^ can be attributed to the stretching vibrations of C–O group. Moreover, the band at 1095 cm^−1^ with relatively high intensity compared to the other ones can be attributed to the stretching vibrations of –C–O–C– groups’ characteristic for polysaccharides. Bands at 953 cm^−1^ and 840 cm^−1^ may have come from the amine group of chitosan [40,41,42,43].

Comparing the spectra of commercial chitosan, it may be noticed that in the spectra of the chitosan-based hydrogels the band of the OH (and NH) stretching between 3000 and 3500 cm^−1^ is almost undetectable. Some reasons of such an observation may be mentioned. Firstly, the mentioned groups form bonds with other functional groups and, in such a way, a crosslinked polymer network is formed. Additionally, as it was mentioned previously, hydrogels, after the synthesis, were rinsed in PBS and such an operation was repeated three times (it was mentioned in Section 2.2). Therefore, it may be supposed that all unreacted reagents were removed from polymer network and the weak signal from the mentioned groups resulted probably from the reaction of OH groups with other groups during the crosslinking process.

For both hydrogels obtained using PEGDA 575 and PEGDA 700, analogous FTIR spectra with absorption bands in the same ranges were obtained. No additional peaks were observed on the spectra of samples containing *Aloe vera* and 10 mL or 12 mL PEGDA 700, respectively. This may be due to the introduction of too little *Aloe vera* in relation to the amount of crosslinking agent, resulting in a strongly crosslinked structure. Inside such a structure of hydrogel material there may be a physical closure of *Aloe vera* juice, so any signals from this substance were not observed. In the case of a sample containing the same amount of *Aloe vera* and 8 mL of crosslinker, two new peaks can be observed, i.e., a medium-intensity peak at 1642 cm^−1^ and a wide low-intensity one between 3300 cm^−1^ and 3550 cm^−1^ with a top at 3435 cm^−1^. The observed peaks can be assigned to the hydroxyl group (3465 cm^−1^) and the stretching vibrations of the COO– group (1642 cm^−1^). The presence of peaks characteristic for *Aloe vera* compounds was also confirmed by Nejatzadeh-Barandozi et al., who, in their analyses, indicated the peak at 3420 cm^−1^ as originating from the hydroxyl group and the absorption band of carboxyl groups at 1640 cm^−1^ [44]. Analogous conclusions are drawn by the works of Lim et al., who stated that the band at 3424 cm^−1^ resulted from the stretching vibrations of –OH group, characteristic for mannose and uronic acid. On the other hand, the band at 1634 cm^−1^ may be also associated with the asymmetric stretching vibrations of –COO carboxylate groups from compounds in *Aloe vera* [45].

### 3.3. Morphological Properties of Hydrogels

**Figure 7:**
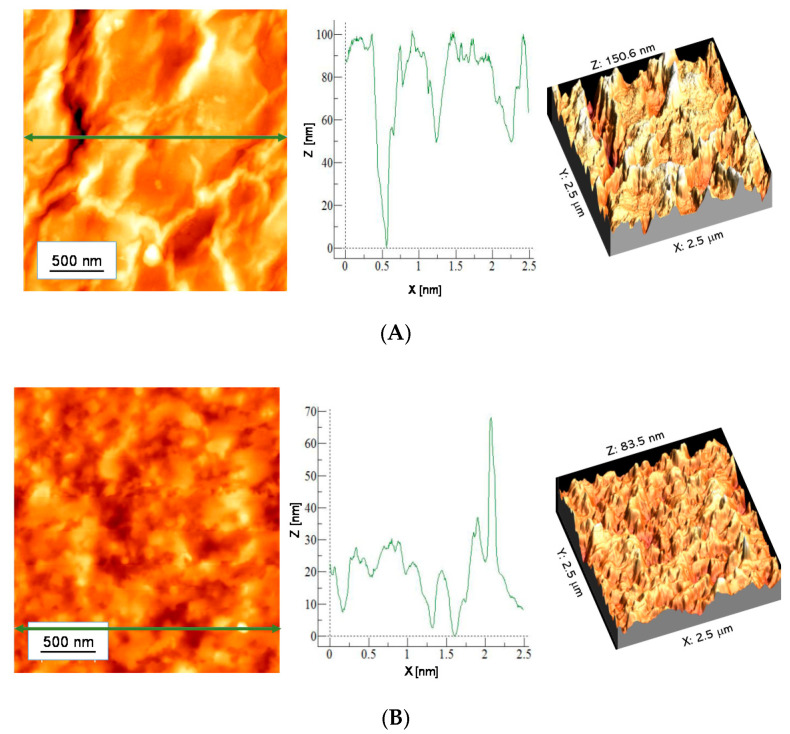
AFM images of unmodified hydrogels: (**A**) 10 mL PEGDA 700, (**B**) 12 mL PEGDA 700.

**Figure 8:**
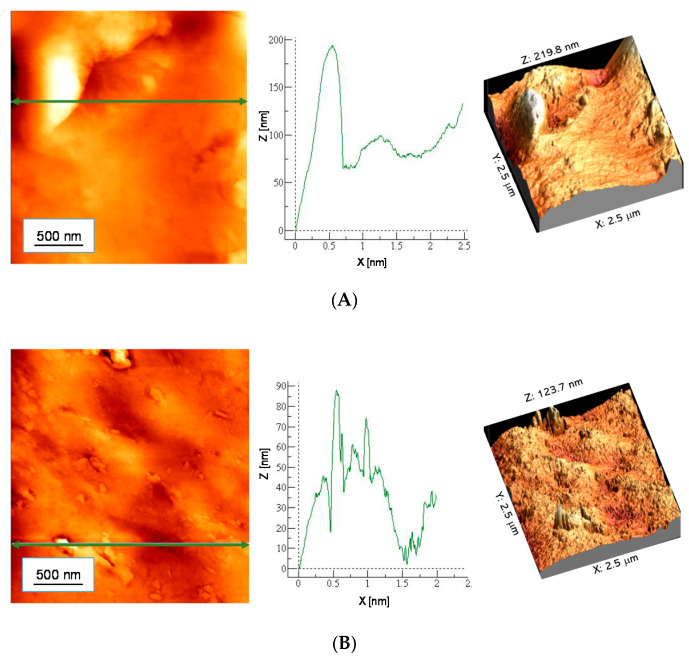
AFM images of *Aloe vera*-modified hydrogels: (**A**) 10 mL PEGDA 700, (**B**) 12 mL PEGDA 700.

AFM images with the material profile along the selected line are presented in Figure 7 and Figure 8. In Table 6 parameters of surface roughness of tested samples are presented.

Nonmodified hydrogels are characterized by a strongly developed specific surface area. In the case of samples modified with 10 mL of *Aloe vera* juice, both for the sample containing 10 mL PEGDA 700 and 12 mL PEGDA 700, a less rough surface was observed. This effect may result from the fact that *Aloe vera* juice is absorbed in hydrogel pores, smoothing its surface.

### 3.4. Sorption Properties of Hydrogels

**Figure 9:**
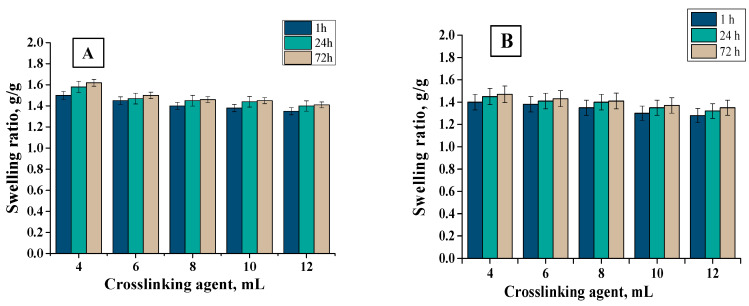
Swelling of materials with different crosslinking agent PEGDA 575 in (**A**) distilled water (average value $\bar{SD}=1.12\%$, number of repetitions *n* = 3), (**B**) hemoglobin (average value $\bar{SD}=1.14\%$, number of repetitions *n* = 3).

**Figure 10:**
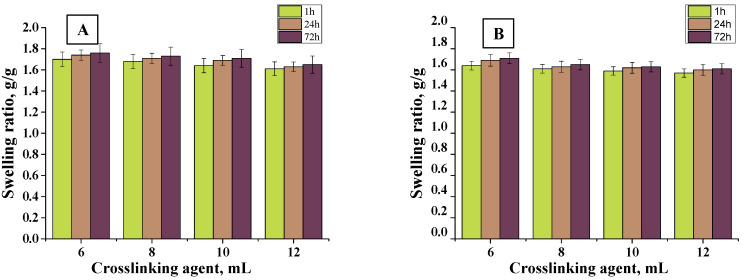
Swelling of materials with different crosslinking agent PEGDA 700 in (**A**) distilled water (average value $\bar{SD}=1.20\%$, number of repetitions *n* = 3), (**B**) hemoglobin (average value $\bar{SD}=1.18\%$, number of repetitions *n* = 3).

**Figure 11:**
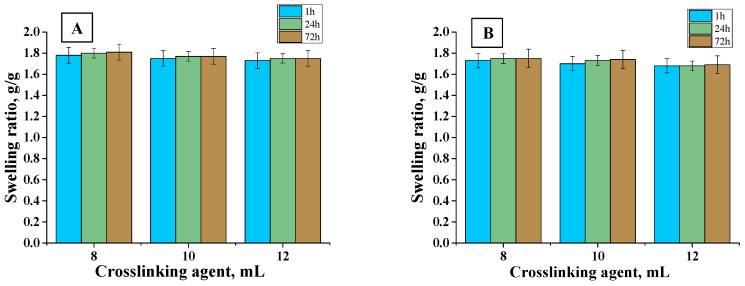
Swelling of materials with different crosslinking agent contents, PEGDA 700 and 10 mL of *Aloe vera* juice in (**A**) distilled water (average value $\bar{SD}=1.11\%$, number of repetitions *n* = 3), (**B**) hemoglobin (average value $\bar{SD}=1.13\%$, number of repetitions *n* = 3).

In order to determine the sorption properties of hydrogels, they were subjected to the swelling analysis. The test was carried out in distilled water and hemoglobin at specific intervals. The results of the analysis are presented in Figure 9, Figure 10 and Figure 11.

Firstly, it may be observed that the tested materials were characterized by a swelling capacity. Hydrogels modified with *Aloe vera* juice and unmodified ones obtained using crosslinking agents of different molecular weights were tested. Swelling ratios of hydrogels with 6, 8, 10, and 12 mL PEGDA 575 were calculated as follows: 1.45, 1.4, 1.38, and 1.35 g/g (distilled water, 1 h), respectively. In turn, for samples with PEGDA 700 it was: 1.7, 1.68, 1.64, and 1.61 g/g (distilled water, 1 h), respectively. All materials synthesized with PEGDA 575 had a lower swelling capacity than those obtained with PEGDA 700. This resulted from the different structure of crosslinked hydrogels. As a result of the use of PEGDA 575, hydrogels in which polymer chains are connected by shorter transverse bonds compared to those formed using a higher molecular weight crosslinking agent (PEGDA 700) were obtained. The difference in the length of these bonds affected the sorption properties of the hydrogels—the shorter bonds, the more compact the material was and, thus, the less fluid absorption. Hydrogels obtained with PEGDA 700 were characterized by higher sorption properties resulting from larger spaces between polymer chains. For all samples, swelling sorption in distilled water was higher than in hemoglobin solution. This was due to the fact that hemoglobin solution contained bivalent ions, which created additional transverse bonds in the polymer matrix. The result was an increase in the crosslinking density of the hydrogel structure and, thus, a decrease in the free spaces available for liquids. Therefore, the greatest swelling was observed in distilled water, where no ions were present. Moreover, hydrogels were characterized by lower absorption of fluids as the amount of crosslinking agent increased. This was also due to the fact that the system became more crosslinked, which increased the network density, thus reducing the sorption capacity, e.g., the swelling rate for hydrogel containing 6 mL of crosslinked agent was 1.7 g/g and for 12 mL was 1.61 g/g (PEGDA 700, distilled water, 1 h). For all samples, the swelling jump after one hour was the highest. Materials containing *Aloe vera* juice were characterized by higher swelling rates than those without this additive, comparing the same swelling medium. This may be due to the fact that once *Aloe vera* was released from the inside of the polymer network, fluids may have penetrated the spaces previously occupied by *Aloe vera* juice, which resulted in higher swelling ratios. The analogous results were presented by Pereira et al., who also used *Aloe vera* as a modifier of hydrogel materials. They suggested that *Aloe vera* addition increases absorption of fluids, which results from hydrophilic properties of *Aloe vera*. As a result, the hydrophilic properties of the hydrogel surface may increase, thus increasing affinity to water solutions and, thus, sorption properties [46].

Sorption ability of material dressings is favorable from the viewpoint of the wound-healing process. The wound exudate that accumulates near the wound may impede this process. Therefore, it is important to develop material that will provide the adequate environment for the wound healing. Investigated materials exhibited good swelling capability. It was proven that the sorption properties depend on the amount and the molecular weight of the crosslinker applied and on the presence of the *Aloe vera* in the hydrogel structure. Such a conclusion is important because it indicates that, depending on the type of the wound (i.e., the amount of the wound exudate), it is possible to use the crosslinker with different molecular weights or its different amount or to modify the hydrogel with *Aloe vera* to obtain the materials with sorption properties adequate to the need of their use.

### 3.5. Mechanical Properties of Hydrogels

**Figure 12:**
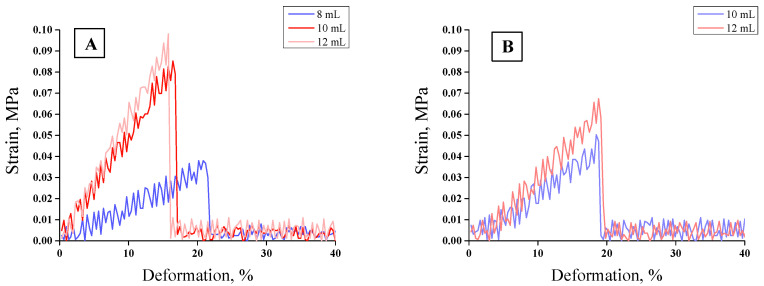
Stress–strain characteristics of hydrogels with different contents of PEGDA 700: (**A**) unmodified, (**B**) modified.

**Figure 13:**
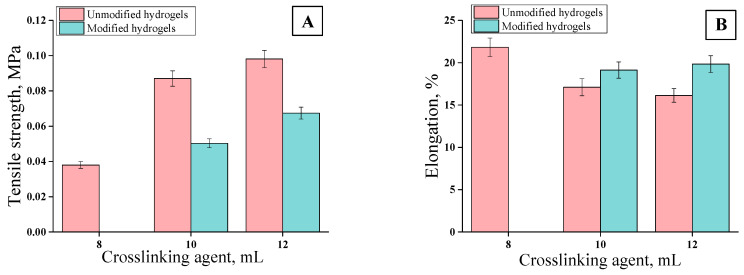
Results of strength analysis of materials modified and unmodified with different PEGDA 700 content: (**A**) Tensile strength (average value $\bar{SD}=1.15\%$, number of repetitions *n* = 3), (**B**) percentage elongation (average value $\bar{SD}=1.17\%$, number of repetitions *n* = 3).

The tensile strength analyses were performed for unmodified hydrogels containing 8, 10, and 12 mL of PEGDA 700 and for modified hydrogels containing 10 and 12 mL of PEGDA 700. The sample containing 8 mL of crosslinking agent was not subjected to the tests due to its too-high brittleness. The results of mechanical investigations of unmodified hydrogels and those modified with *Aloe vera* are presented in Figure 12 and Figure 13.

Strength tests, i.e., the analysis of the deformation to which the tested sample is subjected under the applied stress, were aimed at determining the effect of the amount of the crosslinker used on the elasticity of the hydrogel as well as at evaluating the effect of the additive (*Aloe vera* juice) on this property. Firstly, it may be concluded that with increasing stress, the strain value increased proportionally to the critical point at which the sample was destroyed. An increase in the amount of the crosslinker contributed to the decrease in the elasticity of the hydrogels, as indicated by the decreasing deformation. The crosslinking agent was responsible for the formation of transverse bonds between the polymer chains. With the increase in the amount of crosslinking agent in the reaction mixture, the amount of crosslinks increased. As a result, the material being tested was more crosslinked, its structure was more compact, and, consequently, less susceptible to deformation. As the amount of PEGDA increased, the elasticity of the material decreased while the hardness increased. Material with a higher amount of the crosslinker could withstand higher loads than the material obtained using a lower amount of this reagent. Therefore, such hydrogels, despite lower percentage elongation values, have higher tensile strength values. For example, values of parameters received for unmodified hydrogel obtained using 8 mL PEGDA 700 were as follows: R_m_ = 0. 038 MPa and A = 21.83%. For a hydrogel containing 12 mL of crosslinker, these values were: R_m_ = 0.0981 and A = 16.13%, respectively. Hydrogels modified with *Aloe vera* were characterized by higher elasticity compared to the unmodified ones. In the case of hydrogel modified with *Aloe vera* containing 10 mL of crosslinker, the percentage elongation reached A = 19.13%; whereas for a sample obtained with the same amount of PEGDA without *Aloe vera,* this value reached A = 17.10%. This was due to the fact that the addition of the modifier affected the dilution of the reaction mixture, while at the same time the amount of the crosslinking agent introduced did not change. As a result, the modified hydrogel had a lower crosslinking degree and, thus, had a less-compact structure, which was reflected in its greater flexibility.

In the case of the wound dressings, their favorable properties depend on the place where they are applied. When such a dressing is applied on the wound in a place of high mobility (e.g., elbow, knee, etc.), then the elasticity of the dressing is preferable. Otherwise, it would be difficult to maintain such a dressing. In other cases, the stiffer dressing may be more preferable. Here, we proved that, e.g., by the addition of the adequate amount of the crosslinker it is possible to adjust the elasticity of such a dressing depending on the place of its application. The greater amount of the crosslinker, the more crosslinked polymer structure and the lower elasticity of such formed material. Conversely, the lower amount of the crosslinking agent, the less crosslinked structure and greater elasticity of such a material. More crosslinked polymer structure and the lower elasticity are also related to the decrease of swelling ability (it was proven in Section 3.4. that hydrogels with bigger amounts of crosslinker exhibited lower swelling properties). High swelling properties of the dressing materials for wounds with small amounts of wound exudate are not necessary. Therefore, sometimes bigger attention should be paid to their stiffness. These properties may be modified depending on the needs.

### 3.6. Cytotoxicity of the Hydrogels

**Figure 14:**
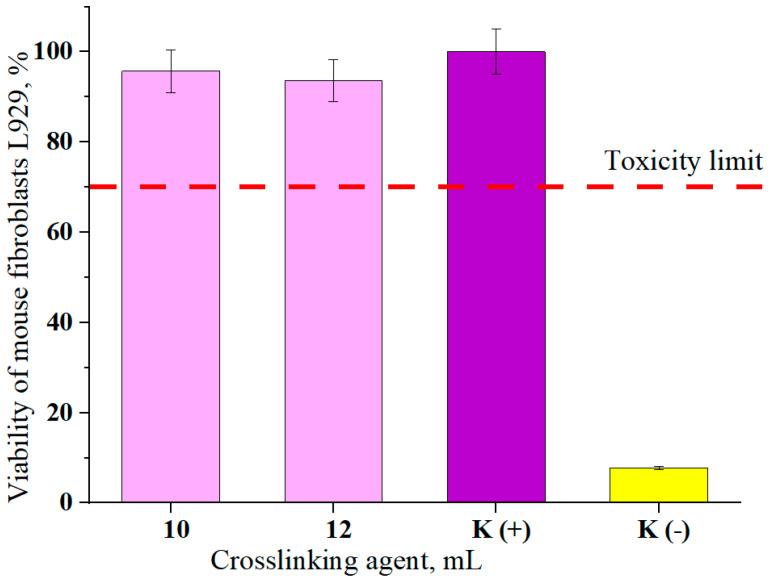
The viability of mouse fibroblasts (L929 cell lines) in the MTT reduction assay (average value $\bar{SD}=1.20\%$, number of repetitions *n* = 3).

In Figure 14, results of cytotoxicity analysis are presented. The viability of mouse fibroblasts (L929 cell lines) was determined in the MTT reduction test according to ISO-10993-5-2009 [47].

The presented results concern the viability of cells incubated with hydrogel materials for 24 h. Hydrogels modified with *Aloe vera* obtained using 10 and 12 mL of crosslinking agent PEGDA 700 were selected for testing because of the presence of the modifying agent and due to the favorable strength properties at the same time. Samples of these hydrogels were characterized by high elasticity determined in mechanical tests. Therefore, if these materials may be potentially used as dressings and biological testing is essential to determine their safety. The basic test is a cytotoxicity analysis. The analysis also included a positive control sample K (+) defined as 100% cell viability. A negative K (−) sample was also determined in which the cells were incubated in the presence of strongly cytotoxic 1% phenol solution. The viability of mouse fibroblasts in the negative control was 7.76%. According to ISO recommendations, the material was considered as cytotoxic when the viability of the cells exposed during 24 h to this material was less than 70%. For the hydrogels samples tested, these values were 95.72% and 93.64%, i.e., above the toxicity limit. Therefore, both samples can be considered as noncytotoxic to mouse fibroblasts (L929 cell lines). Sathiyaseelan et al. determined cytotoxic properties of composites based on fungal chitosan with *Aloe vera* and silver nanoparticles. In his work he showed that the introduction of *Aloe vera* into the composites does not cause cytotoxic properties of such materials and significantly increases cell viability compared to the composites without *Aloe vera* [48]. Therefore, the choice of *Aloe vera* as a modifier of hydrogels is an interesting solution in biomedical applications.

## 4. Conclusions

- FTIR analysis confirmed the presence of characteristic absorption bands from functional groups found in chitosan. For hydrogel samples modified with *Aloe vera*, two new peaks were observed in the wave number: 1642 cm^−1^ and 3435 cm^−1^, which confirmed the presence of this additive in the hydrogel material.
- Samples modified with *Aloe vera* were characterized by higher sorption properties than unmodified ones. In the case of unmodified hydrogels, higher swelling ratios were calculated for samples obtained using PEGDA 700 than PEGDA 575. This dependence resulted from the different structure of hydrogels obtained. A decrease in swelling ratios was observed for samples obtained with a larger amount of crosslinking agent due to the formation of hydrogels with higher crosslinking density.
- Hydrogels modified with *Aloe vera* compared to these unmodified ones obtained with the same amount of crosslinking agent were characterized by greater flexibility. *Aloe vera* application caused increase in percentage elongation of hydrogels’ samples to 23%. Hydrogels containing 10 mL of *Aloe vera* and 10 and 12 mL PEGDA 700 showed the best mechanical properties of modified samples.
- Tested hydrogels did not exhibit cytotoxicity toward L929 murine fibroblasts. Viability of the cells incubated for 24 h with these materials was above 70%.
- Unmodified hydrogels had a well-developed specific surface with a relatively high roughness. The application of *Aloe vera* as a modifying agent caused smoothing of the hydrogel surface.
- The selection of the appropriate conditions for the synthesis of hydrogels allowed us to obtain materials with good strength parameters at the same time with appropriate flexibility. Moreover, these materials did not show cytotoxic properties in relation to the analyzed cells. Due to the above mentioned properties, these hydrogels are an interesting material for biomedical applications, among others, as dressing materials to wounds with difficulty healing.

## Figures and Tables

**Table 1:** Compositions of hydrogels prepared using PEGDA 575 as a crosslinking agent.

1% HD Chitosan in 0.05% Acetic Acid Solution [mL]	Photoinitiator * [mL]	PEGDA 575 [mL]	Name of the Sample
50	0.5	2	2–575
		4	4–575
		6	6–575
		8	8–575
		10	10–575
		12	12–575

* 2-hydroxy-2-methylprophenone, Darocur 1173.

**Table 2:** Compositions of hydrogels prepared using PEGDA 700 as a crosslinking agent.

1% HD Chitosan in 0.05% Acetic Acid [mL]	Photoinitiator * [mL]	PEGDA 700 [mL]	Name of the Sample
50	0.5	2	2–700
		4	4–700
		6	6–700
		8	8–700
		10	10–700
		12	12–700

* 2-hydroxy-2-methylprophenone, Darocur 1173.

**Table 3:** Compositions of modified hydrogels prepared using PEGDA 700.

1% HD Chitosan in 0.05% Acetic Acid Solution [mL]	Photoinitiator * [mL]	PEGDA 700 [mL]	Name of the Sample
50	0.5	8	8–700-a
		10	10–700-a
		12	12–700-a

* 2-hydroxy-2-methylprophenone, Darocur 1173.

**Table 4:** Observation of the course of crosslinking process of hydrogels obtained using crosslinkers with different molecular weights and amounts.

Crosslinking Agent [mL]	Observations for PEGDA 575	Observations for PEGDA 700
2	No hydrogel crosslinking 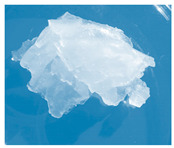	No hydrogel crosslinking
4		
6	Crosslinking/very brittle material → not adequate for mechanical tests 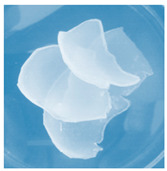	Crosslinking/very brittle material → not adequate for mechanical tests 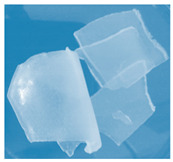
8		
10		The material can be used to prepare samples for mechanical tests 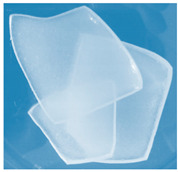
12		

**Table 5:** Observation of the course of crosslinking process of hydrogels containing *Aloe vera* juice obtained using different crosslinkers with different molecular weights and amounts.

Crosslinking Agent [mL]	Observations for PEGDA 700 with *Aloe vera*
8	Crosslinking/very brittle material → not adequate for mechanical tests 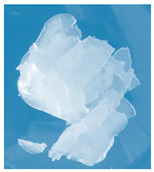
10	The material can be used for mechanical tests 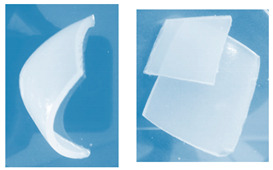
12	

**Table 6:** Parameters of the surface roughness of analyzed hydrogels.

Parameter	Value [µm]
**10 mL PEGDA 700**	
R_z_	34.40
R_v_	14.50
**12 mL PEGDA 700**	
R_z_	44.40
R_v_	27.00
**10 mL PEGDA 700 with *Aloe vera***	
R_z_	91.74
R_v_	52.00
**12 mL PEGDA 700 with *Aloe vera***	
R_z_	27.70
R_v_	17.60

Rz—maximum height of the roughness profile; Rv—maximum valley depth of the roughness profile.
